# CKD.QLD: establishment of a chronic kidney disease [CKD] registry in Queensland, Australia

**DOI:** 10.1186/s12882-017-0607-5

**Published:** 2017-06-07

**Authors:** Sree K. Venuthurupalli, Wendy E. Hoy, Helen G. Healy, Anne Cameron, Robert G. Fassett

**Affiliations:** 1 Renal Services (Toowoomba Hospital), Darling Downs Hospital and Health Service, Toowoomba, QLD Australia; 2 NHMRC CKD.CRE and CKD.QLD, University of Queensland, Brisbane, QLD Australia; 3 Faculty of Medicine, University of Queensland, Brisbane, QLD Australia; 4 Kidney Health Service (RBWH), Metro North Hospital and Health Service, Brisbane, QLD Australia; 5 School of Human Movement and Nutritional Sciences, University of Queensland, Brisbane, QLD Australia; 6 Faculty of Health Sciences and Medicine, Bond University, Gold Coast, QLD Australia

**Keywords:** Chronic kidney disease, Surveillance, Registry

## Abstract

**Background:**

Chronic kidney disease [CKD] is recognised as a global public health problem. Until recently, the majority of information informing on CKD has been generated from renal registries reporting on patients with end-stage kidney disease [ESKD] and on renal replacement therapy [RRT]. There has been a paucity of information on pre-dialysis CKD cohorts, and many issues related to these poorly described populations are unresolved. To this end, international organizations have called for CKD surveillance systems across all countries.

**Description:**

In Australia, we have responded by developing the Chronic Kidney Disease in Queensland [CKD.QLD] with three main platforms consisting of CKD Registry, clinical trials and development of biobank. This registry which is the core component of CKD surveillance was conceptualized specifically for the pre-dialysis population in the public health system in Queensland, Australia. Recruitment started in May 2011, and to date the Registry has evolved as one of the largest CKD cohorts in the world with recruitment close to 7000 patients. The Registry has had many outcomes, including being the nidus for Australia’s first National Health and Medical Research Council [NHMRC] CKD Centre of Research Excellence [CKD.CRE].

**Conclusions:**

The Registry, with its linkage to Queensland Health datasets, is reporting, and is expected to continue generating, significant information on multiple aspects of CKD, its trajectory, management and patient outcomes. Intent of the CKD.CRE is to facilitate an expanded Registry network that has representation from health services, both public and private, across Australia.

**Electronic supplementary material:**

The online version of this article (doi:10.1186/s12882-017-0607-5) contains supplementary material, which is available to authorized users.

## Background

The aim of this report is to describe the structure, progress, update and challenges faced in establishing the first Registry for chronic kidney disease (CKD) in Australia.

Chronic kidney disease, Queensland (CKD.QLD) is the first CKD surveillance system established in Australia [1]. It was conceptualized in 2009 and was developed by early 2011 with CKD Registry, Clinical Trails and Bio-bank as its main platforms. Establishment of CKD Registry was the primary objective based on which other the CKD surveillance activities flow.

Chronic Kidney Disease [CKD] as an entity is a relatively new concept. The National Kidney Foundation Kidney Disease Outcomes Quality Initiative™ [NKF KDOQI™] developed the CKD definition using the estimated glomerular filtration [eGFR] and proposed CKD staging, which included a continuum of kidney dysfunction ranging from mild damage to more severe forms of kidney failure requiring renal replacement therapy [RRT] [2].

Despite an estimated worldwide prevalence of 10 to 15%, making CKD one of the most common non-communicable diseases [NCD], neither the World Health Organization [WHO] nor United Nations [UN] General Assembly recognized CKD as an important NCD in their reports [3, 4]. Part of this lack of recognition may be because information to date has been provided by renal registries characterizing the population on RRT due to end-stage kidney disease [ESKD] [5–7]. This information, combined with output from other research studies, has been the basis for the majority of clinical guidelines published by various national and international organizations [8, 9].

CKD is a major determinant of public health in any country and is associated with increased morbidity and mortality, predominantly cardiovascular disease [10, 11]. CKD directly contributes to 10% of deaths in Australia and is a huge economic burden on healthcare systems [12]. Kidney damage also interacts with, and influences poor health outcomes, of other major NCDs [13]. It has also been established that strategies such as lifestyle changes, blood pressure control, diabetes management, usage of renin-angiotensin system inhibitors [RAAS] and manipulation of metabolic parameters have an important role in delaying the progression of the disease [14–16].

There are still many unresolved questions with regards to the definition, management and follow up of people with CKD. Issues related to estimation of prevalence of this pre-dialysis cohort, limitations of widely used eGFR estimation equations, implications of the age related decline of eGFR, incorporation of albuminuria determination as an important marker of kidney damage and progression in primary practice and the role of community screening and timing of referral to nephrology services are still debatable [17–21]. Even now many developed countries have no sustainable mechanisms of managing this large pre-dialysis CKD population [22].

Recognizing the need for a better understanding of the burden of CKD, in 2006 the International Society of Nephrology [ISN] organized a major meeting of experts. The Kidney Disease Improving Global Outcomes [KDIGO] Controversies Conference advocated for screening and surveillance of CKD stage 3 to 5, and preferably all stages [23].

The situation in Australia is no different from rest of the world with traditionally RRT-cohort data available from the Australia and New Zealand Dialysis and Transplant Registry [ANZDATA] and estimated CKD prevalence rates from the Australian Diabetes, Obesity and Lifestyle Study [AusDiab Study] with no systems to study and define the pre-dialysis CKD population [7, 24]. This limitation has been noted in the major works and publications produced by the Australian Institute of Health and Welfare [AIHW], which have been largely dependent on ANZDATA and other hospital based information [25].

Responding to the International call for CKD surveillance and the need for a sustainable CKD surveillance program, Chronic Kidney Disease in Queensland [CKD.QLD] was conceived. The initial steps involved in establishing first CKD surveillance system in Queensland were described earlier [1]. The Registry was the main and core component of the CKD.QLD surveillance. Here we describe the establishment, progress and update of the CKD Registry; the other two platforms (clinical trials and the Bio-bank) will be reported independently. We hope that this paper would be of help to any group in the world proposing to develop such registry by identifying the scope and challenges.

## Construction and content

### The concept

The idea of CKD.QLD was conceived in September 2009. Queensland has a population of about 4.8 million, distributed in large metropolitan areas, smaller cities and towns, rural settings and very remote regions. It has a rich mix of socioeconomic status, great ethnic diversity with many migrants, a large population of retirees [over 65 years of age], and a population of 155,825 [3.6%] Indigenous Australians [26]. Queensland Health [QH], through which most Queenslanders receive health care, is the sole public health provider. Significant numbers of the population receive healthcare through the private sector also. This profile of Queensland mirrors the national picture, suggesting that data and outcomes from CKD.QLD will reflect the magnitude of the Australia-wide CKD burden.

CKD.QLD was established as a collaborative of all the public sector renal practices in Queensland. The collaborative embraces a multidisciplinary panel of investigators, including nephrology, nursing, allied health, primary care, Indigenous health, molecular bioscience, information technology, and health economics. The Registry formed the backbone of all future research activities of CKD.QLD.

### The objectives of the registry

**Fig. 1:**
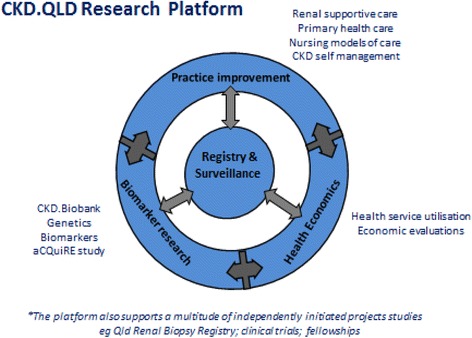
Research platforms of CKD.QLD Registry

The main objectives of Registry are to profile patients with CKD and their management in Queensland, starting with those in public renal practices, by establishing and maintaining a database and registry of CKD patients in the state. This registry and database records current and longitudinal patient outcomes and clinical practice patterns, and informs CKD health practitioners, QH, national groups such as the AIHW and most particularly CKD patients. It is expected that the information available through the CKD Registry will have synergistic flow to ANZDATA. The scope of the research platform of CKD.QLD is enabled by the Registry, which is central to informing all streams of affiliated research enquiry (Fig. 1).

### Why a CKD registry?

Patient registries have been defined as “an organized system that uses observational study methods to collect uniform data [clinical and other] to evaluate specified outcomes for a population defined by a particular disease, condition, or exposure, and that serves a predetermined scientific, clinical, or policy purpose [s]” [27].

Cohorts of pre-dialysis CKD patients have been established for the purpose of longitudinal study and analysis of outcomes, such as the Chronic Renal Insufficiency Cohort [CRIC] study [28]. Similar cohorts developed across the globe to improve the knowledge base of CKD management [29]. However, a cohort study is less flexible and is limited by its predefined objectives and aims. Such studies have limited numbers of recruitment and may exclude certain populations from the study by design. Alternatively, data from patient registries, which can include demographic and clinical information, as well as longitudinal outcome measures, may resemble traditional observational cohort studies.

However registries differ from such cohorts in multiple ways. Registry-based studies can be flexible in the scope and focus of the data collection and different mechanisms can be incorporated over time to address additional needs. New studies can be initiated within an ongoing registry during its course, and the database derived from the registry may be used to support secondary studies, such as those that link the registry database with other data sources to explore new questions or derive new outcomes [30].

A patient registry has multiple functions. The longitudinal data collected throw light on the course of disease and treatment options and their outcomes. Registry data can also be used to generate prediction factors influencing the prognosis, care patterns and disparities in the delivery of care. Variations in patient characteristics and outcomes based on demographic data including geographical location, educational status and socioeconomic conditions can also be evaluated. Finally, comparison of data across different healthcare providers may provide an opportunity to assess quality improvement [31, 32].

Information gathered from these registries become useful not only for traditional stakeholders such as the medical profession and hospitals, but also for those determining health care policies and effective utilization of resources. Data from registries can also be used to evaluate various models of care [MOC].

### Initial steps in the development of the registry

As noted, the concept of CKD.QLD was initiated in September 2009. To establish a state framework, a survey of CKD practices and facilities in Queensland was conducted in early 2010 [33]. This survey identified the level of CKD care in QLD both in private and public facilities and provided an estimate of the workforce and resources available. During this survey it became clear that there was a significant overlap of CKD population across public and private sectors and hence it was decided to focus on the public sector initially. This first survey was followed by a web based questionnaire [Survey Monkey] exploring CKD management across all the public sector renal services in Queensland [34]. Senior management comprising both medical and nursing personnel responded to the survey, providing their view of the existing patterns of CKD care. It was evident that most patients [90%] were seen by nephrologists and that availability or use of allied health support was variable, and was largely utilised within larger metropolitan renal services. The survey suggested that there were about 10,000 CKD patients in these practices, providing an estimate of the population who could potentially be recruited to the registry. These two surveys also outlined the existing resources and pre-identified potential areas for initiatives and improvements in management of people with CKD in Queensland.

### Data collection and management

In July 2010 a data management workshop was conducted to define the process of data capture across various renal services. This workshop involved a spectrum of stakeholders including medical, nursing and allied health. It was apparent that most of the units were engaged in some form of data collection. Although a couple of centres have web based data entry, other units collected data in excel form [Microsoft]. It was decided to develop a central data repository [CDR] to collect and collate all data, with that function based at the Centre for Chronic Disease, University of Queensland, Faculty of Medicine in Brisbane, Queensland. All units contributed data to the CDR with larger metropolitan centres reporting at 3 monthly intervals and smaller regional centres once every 6 months.

### Ethics submission and approval

In late 2010, a multi-centre ethics application incorporating all 12 QH renal services was submitted to the Queensland Health [QH] Human Research Ethics Committee [HREC Approval: HREC/10/QHC/41]. With granting, site specific approvals were then obtained at these 12 services over the next 12 months. In 2014 and 2015, two additional renal services received approval to join the Registry, including Inala Primary Care, a primary care practice that provides a CKD program. In parallel to Queensland Health ethical oversight, an ethics application was submitted to The Medical ResearchEthics Committee [MREC] of The University of Queensland, which was also approved [2011000029].

With the closure of the Queensland Health Central Ethics Office in 2015, the CKD.QLD Registry was transferred to the Royal Brisbane and Women’s Hospital Ethics Committee [HREC/15/QRBW/294]. The Registry protocol stipulates patient inclusion via an informed consent process. A “waiver of consent” was explored with a request to Ethics for consideration, but this was not granted. Recruitment to the Registry began in May 2011.

### Financial resources and funding for the registry

The initial funding for CKD.QLD Registry was supported by an untied grant from Colonial Foundation, Melbourne, Australia (W. Hoy 2008–2011) and an untied NHMRC Australia Fellowship to W. Hoy (2008–2013, # 5110810). This effort was also supported by unrestricted grant from AMGEN, Australia and in-kind support from Queensland Health. The ongoing funding is now incorporated in the surveillance stream of the NHMRC Centre for Research Excellence in CKD (CKD.CRE) (W. Hoy et al, 2015–2019, # 1079502). The long-term success of Registry would largely depend on the ability to recognize and incorporate registry activities into public health system as an important core component of healthcare delivery.

### Management and administration of the Registry

The overall functioning of the Registry is supervised by Steering Committee Chaired by Professor Wendy Hoy. The registry activities are very much entrenched into the NHMRC CKD.CRE surveillance stream. However, the day to day functions of the registry are managed by a project manager (0.6 FTE) and senior biostatistician and data manager (1.0 FTE). There is an ongoing significant in-kind contribution from renal fraternity from QH.

### Data sources

The Registry uses a data linkage framework which centralises data captured by multiple mechanisms to an individual patient via a unique identifier. The linkage framework can include additional data sources as they become available and as indicated.

### Queensland health - all patients: “the Viewer” platform

The Viewer is a read-only web-based application that displays consolidated clinical information sourced from a number of existing Queensland Health enterprise clinical and administrative systems. Via patient unique identification, The Viewer summarises current health status [alive or deceased], and patient demographics. In addition, The Viewer provides unique access of a single patient’s [Queensland inclusive] public health hospital admissions and incorporated discharge summaries. The Viewer also contains an access portal to all public pathology and radiology results, procedures performed and medications prescribed [35].

### Integrated electronic medical record (ieMR)

In Queensland, patient medical records have historically been paper based, limiting access to information with records also limited to a single health care facility. Since 2007, Queensland Health has been transitioning to an electronic data (eHR) platform of integrated electronic Medical Records, supporting a single view of a patient’s medical record, and making it easier to share information about the patient’s history, and thus increasing quality of care and providing for a sustainable health system. From a renal perspective, the ieMR solution has been rolled out in selected services, but of those now digital, ieMR provides an invaluable Registry data linkage and data verification platform.

### Pathology Queensland - all patients, all public pathology results

Pathology Queensland provides pathology services to all Queensland Health public hospitals. Pathology Queensland is composed of a hierarchical, networked system of 34 laboratories that consist of district laboratories in rural hospitals, group laboratories in large regional hospitals and unit base laboratories providing tertiary referral services in the metropolitan teaching hospitals. All patient pathology results are available for review with ethical approval [36].

### Unit specific data capture mechanisms

#### Cairns and hinterland hospital and health service – Cairns hospital renal service

Audit4: Developed by Software 4 Specialists [S4S], Audit4 is a clinical application devised specifically for medical and surgical specialties [37]. Utilised in Cairns [the only Queensland public site using this system], Audit4 captures electronic prescriptions, electronic filing of pathology results, correspondence to referring doctors, procedure reports, and CKD-specific and procedure-specific worksheets. Combined with other Audit4 users across Australia it provides an additional mechanism of data linkage at national level.

#### Townsville hospital and health service – Townsville and Joyce palmer [Palm Island] renal health service

**Ferret**: Ferret is a primary health care patient information and recall system [PIRS] used within Queensland Health. It has the capacity to capture a wide range of information to assist staff care for clients and manage the services they provide. It is in use in over 80 sites throughout Queensland, though only in Townsville from a renal service perspective. Ferret is configured in line with the Chronic Disease Guidelines and best practice and uses include Whole-of-life Care Plans, Facility Practice Management and Appointments, a Chronic Diseases Register, and modules for Service Activity and Clinical Reporting, Health Education and Promotion and Capturing information for State and Commonwealth Funding Reporting. Ferret provides both demographic and clinical information from all clients who come in contact with a health service to determine individual life-long health care plans, and provides the consenting baseline and longitudinal information for these Registry patients.

#### Metro north hospital and health service – Royal Brisbane and Women’s hospital kidney health service

The Renal Data Base, the “Big R”. Developed as an in-house portal, this is the data source for patient consents and demographics, RBWH identifiers, renal data, and renal outcomes. It collates all renal letters - documenting patient course and treatment, and has the ability to link and download both public and private pathology reports.

#### Metro south hospital and health service – Logan renal service and Princess Alexandra hospital nephrology and Transplant service

**MINTS** [**M**etro south & **I**pswich **N**ephrology & **T**ransplant **S**ervice] data base: This service has developed an in-house database which is a comprehensive patient platform across all models of care. From a Registry perspective, the portal provides those patients who have consented, together with demographics, hospital identifiers, selected renal data, and patient outcomes.

**ERIC:** is a hybrid electronic medical records management system that provides secure access to information used to deliver individualised patient care across the Logan-Beaudesert Health Service District. ERIC combines scanned images, direct data entry and interfaced information including all renal letters documenting patient course, treatment, outcomes and education/decision making.

### Excel spreadsheets

**Fig. 2:**
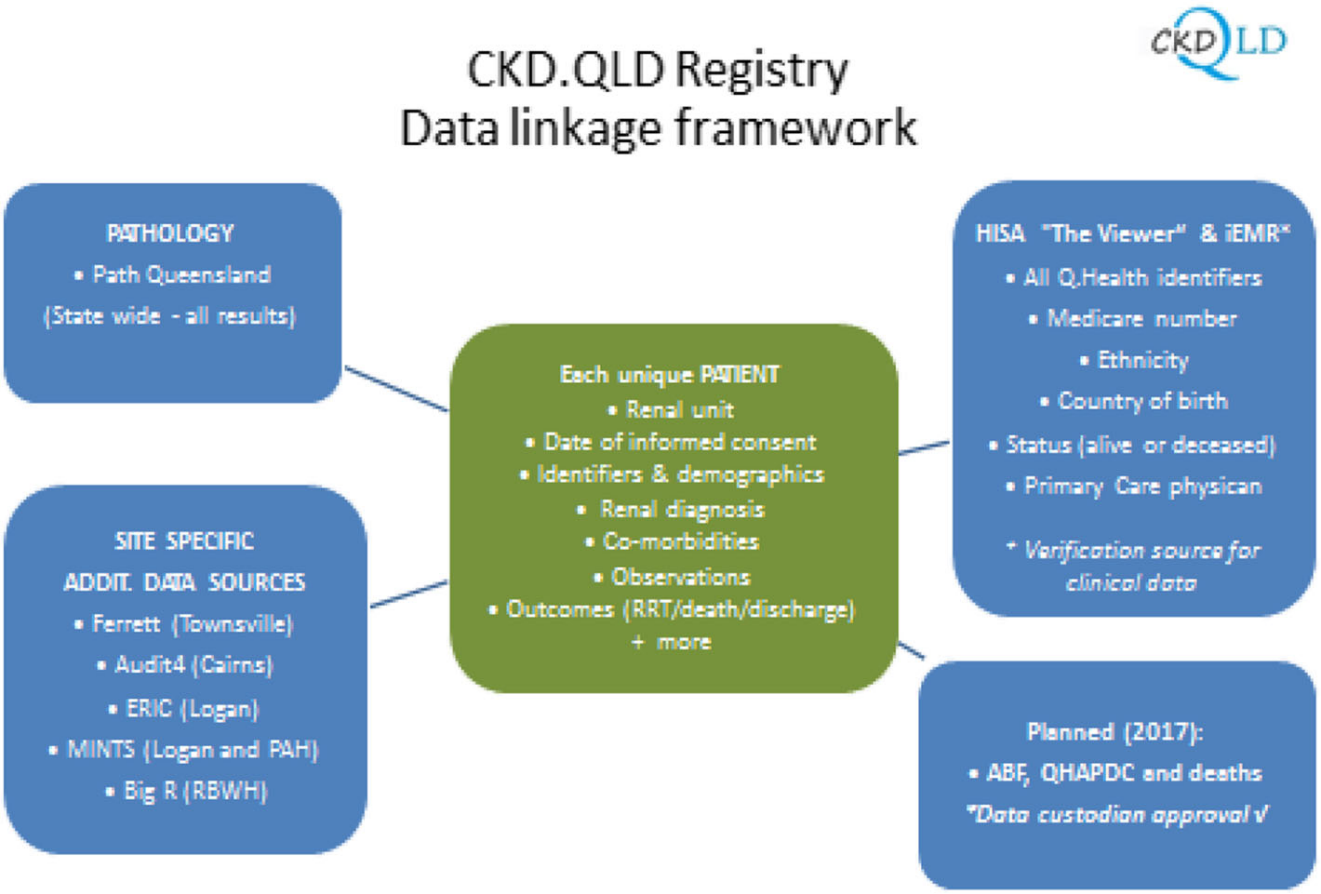
Data linkage networks of CKD.QLD Registry

Other sites without an established CKD data capture mechanism have developed service specific excel spreadsheets which are used to track patient consent, demographics and renal data information. Some of these data sources are summarised below: (Fig. 2).

### Data linkage

In February 2017, and building on the established CKD.QLD data linkage framework, a Registry sub-study ethics application was submitted and approved. This study aims to go a step further in characterising patients with CKD, and to provide a comprehensive view of the true health services utilisation, costs and outcomes of people with CKD. Data for this expanded view will be provided through collaboration with Queensland Health data Custodians for Queensland Hospital Admitted Patient Data Collection (QHAPDC), Death, and the Activity Based Funding [ABF] Model Output data. This state-wide data linkage program will be enabled by the Statistical Services Branch, Department of Health, and with a Public Health Act application and approval. In summary, these approaches reflect the changing dynamic of data capture mechanisms in the public health clinical renal environment. A strength of the CKD.QLD Registry is its ability to grow in-line with the evolving electronic health record (eHR) platforms across the State and nationally**.**

### Confidentiality and protection of data

The information related to these participants is stored at the CDR located on the Herston campus of The University of Queensland, and under the mandate of the Registry’s Principal Investigator, Prof Wendy Hoy. The cohort data are stored electronically with an encrypted and password protected file. Although internally identifiable and re-identifiable, the health information related to participants is managed within a secure framework of patient confidentiality. At no time is information released or reported that is identifiable to a unique individual. The CKD.QLD registry is managed as per legislated requirements including the Information and Privacy Act 2009, and as per the National Health and Medical Research Council [NHMRC] 2007, National Statement on Ethical Conduct in Human Research [updated May 2015] [38].

### Data reporting

The Registry data are analysed by researchers using Statistical software. This data are converted into reports of de-identified data for stakeholders and collaborators. The information is of interest to Queensland Health, the Australian Institute of Health and Welfare [AIHW], The University of Queensland, the Australasian and New Zealand Society of Nephrology and Kidney Health Australia. Outcomes are published in academic journals and on the CKD.QLD website: **www.ckdqld.org**, for professional and patient information and dissemination. In addition, individual site reports are developed at intervals to optimise potential generalisability. A minimum recruitment goal to trigger the first report for any site was originally specified as 25% for larger sites and 50% for smaller sites: this remains flexible, in the interests of feedback and accountability, but more desirable targets are probably 50% and 75% respectively.

### Data access

CKD.QLD facilitates data usage for variety of reasons including quality assurance, clinical audits, surveys, reports and research projects. The Registry actively encourages communication from anyone interested to send requests and all requests would be considered positively provided they fall within the guidelines and ethical parameters of the Registry. (Additional file 1)

### Methods of data collection

All adults over 18 years with a diagnosis of CKD, able to provide informed consent, and attending public renal clinics in Queensland Health facilities, are eligible to be approached for recruitment to the CKD.QLD Registry. The diagnosis of CKD was based on international guidelines [2]. Patients on RRT are not included, as all RRT patents are profiled in the ANZDATA registry. However, once enrolled, CKD.QLD participants can be followed through and after the institution of RRT if that occurs. Similarly patients with acute kidney injury (AKI) are excluded unless they developed CKD subsequently and met the diagnostic criteria for CKD. Demographic and clinical details are captured incrementally in various data formats as described above. Demographic variables recorded include age, gender, ethnicity and geographical location, identified by postcode.

Renal function is classified according to estimated glomerular filtration rate [eGFR], calculated according to the international guidelines as applicable in Australia using the MDRD formula from 2005 until September 2012 and the CKD EPi formula from October 2012 [39, 40]. Proteinuria/albuminuria is defined by availability of any or all the pathology reporting mechanisms: urine albumin: creatinine ratio [uACR], protein: creatinine ratio [uPCR], 24 h urinary protein and/or positive urine dipstick test for protein. Based on the level of these results they are then categorised into three groups- normal, microalbuminuria and macroalbuminuria. The primary renal diagnosis and or any additional renal diagnoses, as assigned by the nephrologists caring for the patient, [determined through a combination of history, clinical examination and investigations such as ultrasonography, urine analysis, blood chemistry and serological studies, and sometimes histological diagnoses] are documented. The renal diagnosis is mapped to ANZDATA codes, allowing comparative analysis with the ESKD Registry [41]. Where ANZDATA codes are not available for specific disease diagnosis, new CKD codes are assigned as an alternative to the generic code of 0 for “Other”. Hypertension as defined by a blood pressure [BP] recordings of >140/90 mm of Hg on two separate clinic visits, or a previous history of hypertension and/or prescription of antihypertensive medications. A diagnosis of diabetes is based on a history of DM, HbA1C more than 6.5 on two occasions or treatment for diabetes according to Australian Diabetic Society (ADS) guidelines [42]. Other information related to diabetes including impaired glucose tolerance and gestational diabetes is noted. Concomitant presences of vascular co-morbidity including cardiac, cerebral and peripheral vascular disease are included in the data. Other major co-morbid conditions including but not limited to chronic lung disease, sleep apnoea, gout, cancer, psychiatric illness and cognitive impairment are also noted.

Weight in kilograms and height in metres and body mass index [BMI] calculated on the date of consent are also recorded. Obesity is expressed according to BMI, based on WHO criteria adopted by department of Health, Australian Government [43]. Smoking status is classified as former, never smoked, current smoker or unknown. Pharmaceuticals prescribed including RAAS blockers and lipid lowering agents are noted. Data are censored when exit outcomes are reported, and include initiation of renal replacement therapy [RRT], death, discharged from service or transferred out to another renal facility.

### Statistical analysis

All data analyses are undertaken using Stata; currently Stata Corp. Stata Statistical Software: Release 14.0, College Station. TX: StataCorp LP, 2015 [44].

## Utility and discussion

### Recruitment progress

**Fig. 3:**
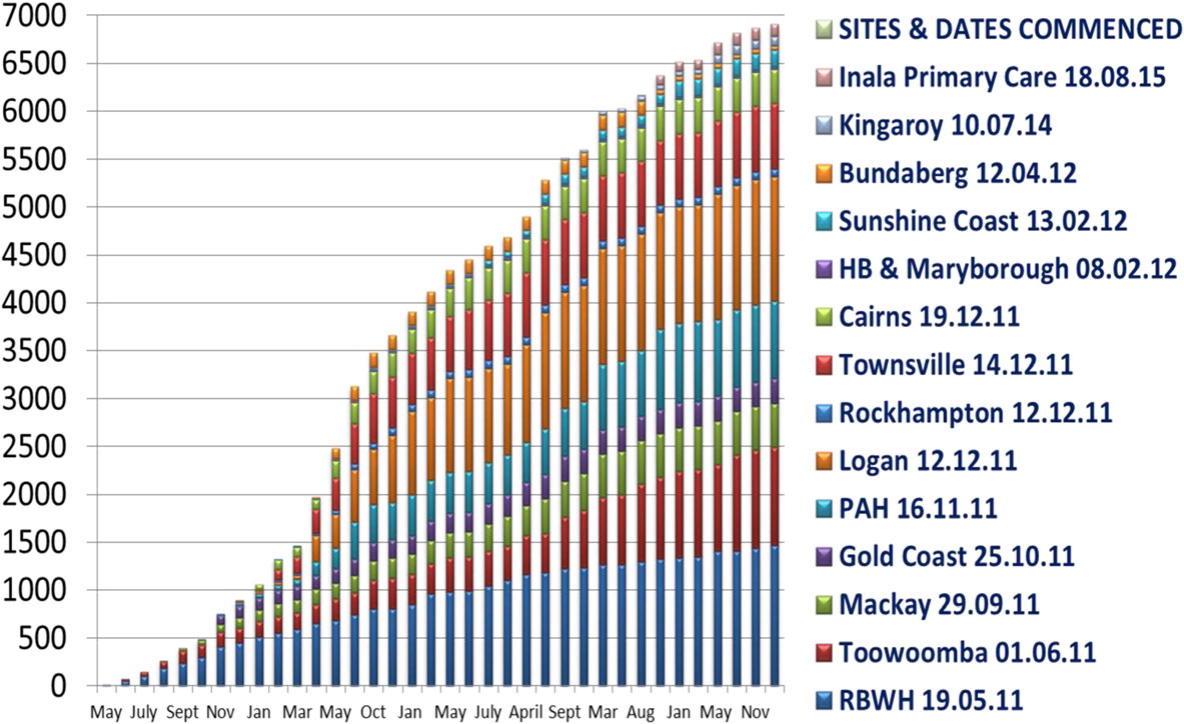
Enrolment with consent to CKD.QLD Registry [6991] Active and Inactive

Recruitment has progressed since May 2011 across the multiple centres. At time of authorship, the Registry has 6900 patients inclusive of those who consented and are now deceased or on an RRT. The majority of patients have been recruited from four major centres, with recruitment of their prevalent CKD patients ranging from 30% to 98% (Fig. 3).

The main impediments to recruitment have been identified as lack of resources at participating services and sites, particularly those to support the processes of informed consent. Refusals are rare, [<0.5% of people approached], and there have been only two cases of formal withdrawals from the registry. Individual site reports have been generated for 10 of the 14 services who have met the qualifiers for reporting [representative recruitment targets as discussed previously].

### Major strengths of the registry

Although short of achieving recruitment targets across all sites, CKD.QLD Registry has evolved as one of the largest CKD surveillance cohorts in the world [45]. This level of recruitment has permitted descriptive assessments of CKD; of demographic features, of age and CKD, BMI and CKD, co-morbidities and CKD, cardiovascular disease and CKD, AKI and CKD, and limited description of ethnicity and socioeconomic status with CKD. They permit assessment of CKD progression and of morbidity, mortality and RRT and predictors of such outcomes, and potentially of comprehensive hospitalisations and health service consumption. The data also permit a comparative analysis of the CKD population in Queensland with those on RRT derived from ANZDATA. Important research initiatives also include medical, nursing and allied health contributions. Nursing and allied health projects revolving around alternate models of care [MOC] and nutritional assessment are at a critical stage of reporting [46].

**Fig. 4:**
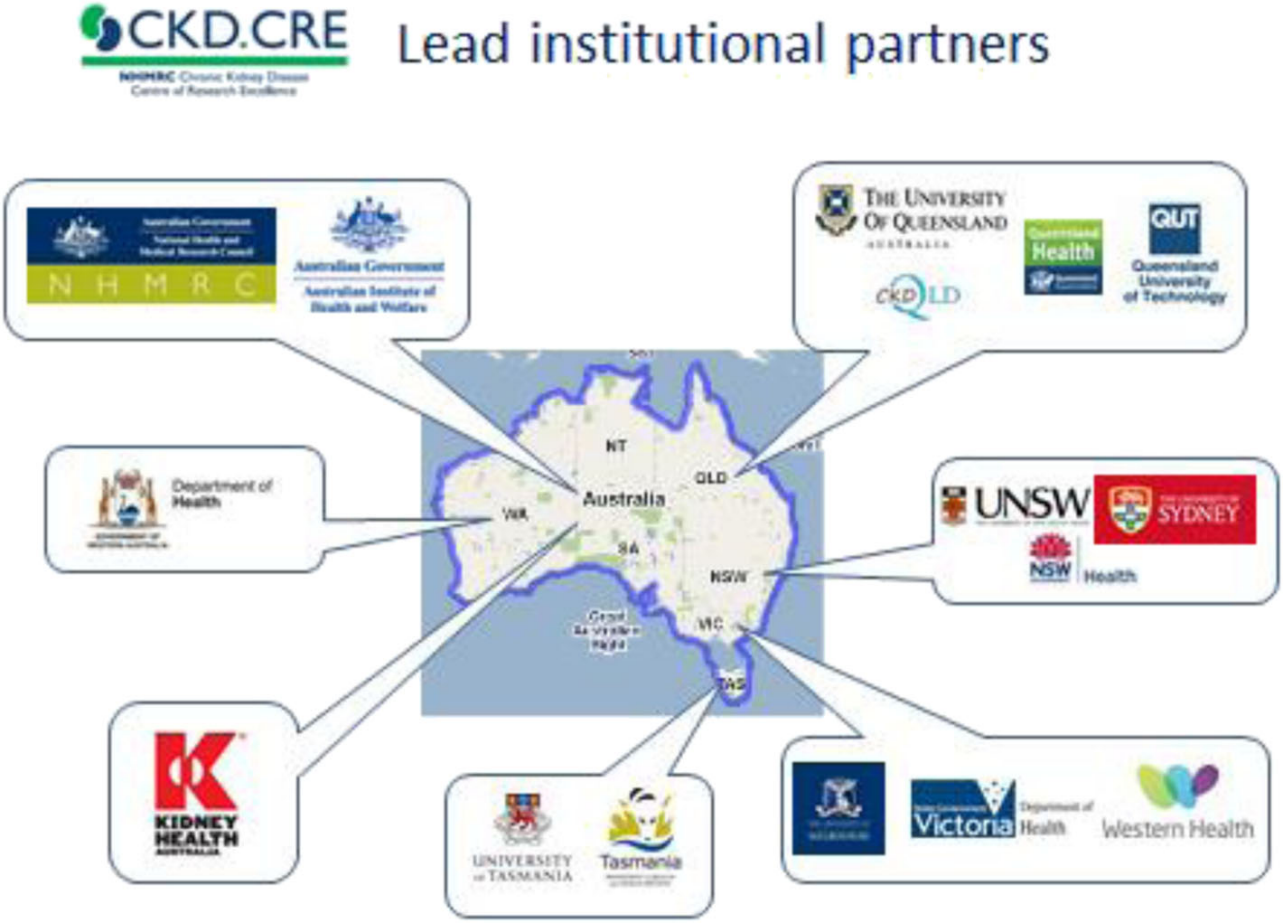
Chronic Kidney Disease Centre of Research Excellence (CKD.CRE) Network

Another most important outcome of CKD.QLD is the establishment of NHMRC funded CKD.CRE. The CKD.QLD Registry provided the core research framework [expanded to the national stage under the CRE] and facilitated the collaborative structure of this important initiative. The CKD.CRE has distinct predefined objectives and research streams, which in many ways nationalise the central themes of CKD.QLD through a network of national collaborations shown: (Fig. 4).

### Limitations of the registry

The Registry data, representing 60% to 70% of the estimated target population, are limited to the patients with CKD referred to renal services in public hospital system. Barring significant overlap of care between public and private systems, CKD patients exclusively managed in private practice were not included in the registry. Moreover, penetration into primary care and general practice are required to define the extent and magnitude of the CKD problem in those environments. CKD patients who are managed in primary care alone were not represented in the Registry. The most recent Registry-affiliated service, Inala Primary Care, is evaluating a service model of empowerment of general practitioners in CKD management and may change the picture to certain extent.

### Future challenges

A major challenge is resourcing recruitment via informed consent, and supporting the teams who gather, manage, analyse and generate the data. That is all externally sourced at this point, and on the premise that these units are participating in research. In the long-term the continuing success of the Registry depends largely on incorporating the concept of Registry as a QH service initiative and being part of everyday routine renal practice. Also the products generated through the Registry must make the case to healthcare provider [QH] that such surveillance is very important: for predictions, for policy, for planning, including models of care and health economics. Ultimately the case will need to be made that the system helps to save heath costs by reducing rate of dialysis, development of alternate MOC, establishment of renal supportive care systems and by provision of telemedicine while not compromising QOL or outcomes. Another challenge is overcoming the tendency to interpret renal outcomes in the dialysis framework. This requires a major cultural shift in the way renal medicine is practiced and viewed by physicians and by the public. Multiple disciplines are in the process of developing surveillance systems to delineate the extent of their respective issues and manage disease conditions effectively. All these efforts require resources and funding for long-term survival. Establishment of an integrated model of registries, as part of routine clinical practice, would, suitably modified, serve the needs of several disciplines across chronic disease management thus avoiding duplication of work and reduce wasteful expenditure.

## Conclusions

CKD.QLD Registry is the first pre-dialysis CKD registry in Australia. It has evolved over 6 years to be one of the largest CKD registries, enabling generation and comparison of outcome data across many such initiatives in the world. The registry, with recruitment close to 7000 participants, has provided major impetus to multiple research initiatives across multiple streams involving medical, nursing and allied health. Its ability and scope to link with Queensland Heath data systems is expected to equip policy makers to identify potential areas of change and improvement.

## Additional file

Additional file 1: CKD.QLD Registry data access guidelines –updated August 2016. (PDF 509 kb)

## Abbreviations

ANZDATA: Australia and New Zealand dialysis and transplant registry
BMI: Body mass index
CDR: Central data repository
CKD.CRE: Chronic kidney disease centre of research excellence
CKD.QLD: Chronic kidney disease, Queensland
eGFR: Estimated glomerular filtration
NCDs: Non-communicable diseases
RRT: Renal replacement therapy
uACR: Urine albumin: creatinine ratio
uPCR: Protein: creatinine ratio
