# Supplementary material for: CKD.QLD: establishment of a chronic kidney disease [CKD] registry in Queensland, Australia
Source: BMC Nephrol. 2017 Jun 7;18:189. doi: 10.1186/s12882-017-0607-5 (PMC5463396; doi:10.1186/s12882-017-0607-5)
Supplement: Additional file 1: — CKD.QLD Registry data access guidelines –updated August 2016. (PDF 509 kb) [file 12882_2017_607_MOESM1_ESM.pdf]

▪ CKD.QLD Registry

HREC Reference Number (MNHHS): HREC/15/QRBW/294 as of June 2015
HREC Reference Number (OHMR): HREC/10/QHC/41 - Nov 2010 to June 2015
University of Queensland Medical Research Ethics Number: 2011000029

Approved 17 November, 2010. Queensland Health Central Office Human Research Ethics Committee.
"The objectives of CKD.QLD are to profile Chronic Kidney Disease (CKD) patients and their management in Queensland, starting with those in renal practices, by establishing and maintaining a database and registry of CKD patients in QLD. This registry and database will record current and longitudinal patient outcomes and clinical practice patterns and will inform CKD health practitioners, Queensland Health, national groups such as the Australian Institute of Health and Welfare and most particularly CKD patients."

▪ CKD.QLD Governance Structure. Chair: Prof Wendy Hoy

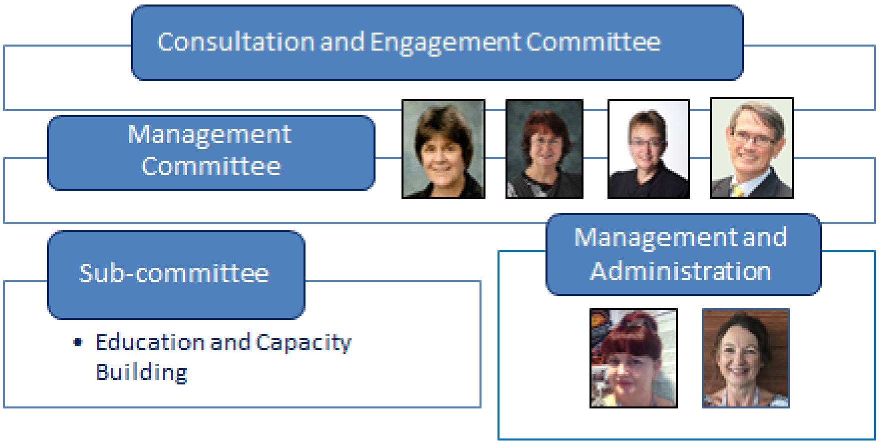

▪ Data Access Guidelines

CKD.QLD encourages use of its data for a variety of purposes, including quality assurance, audit, surveys, reports and research projects, and we welcome enquiries as to use and interpretation of the information we collect. We encourage communication with the Registry to help us to meet your request, particularly if you have not used Registry data before. Provided they fall within our guidelines and the ethical parameters of the Registry, simple requests for tabular data are usually dealt with within 2 weeks; more complex requests may take a little longer.

Request Guidelines. Data requests will be considered within three broad headings:

1. Nature of request

The request should include the information sought, the usage proposed (including further analyses if appropriate) and an undertaking that the source of the data will be acknowledged. An indication of the level of the data requested should be given. (Extensive analysis of case-level data would normally be undertaken as part of a collaborative effort with the Registry.) We may also provide advice about the methods of analysis, or may suggest other sources of advice to ensure the best use is made of the data.

2. Nature of requesting party

In general, requests from Queensland Health renal physicians, renal unit staff, charitable bodies, academic institutions, other registries and government departments are fulfilled without charge. Individual hospital outcomes will usually only be released to people from that institution with the approval of the head of the contributing department. Requests from industry and other bodies are considered on a case-by-case basis, and may be subject to a processing fee.

Version 3.0 August 2016. Review date: August 2017.
ACKNOWLEDGEMENT:
The ANZDATA Registry Data Access Guidelines where benchmarked in the development of this document.

3. Patient Confidentiality/Privacy Regulation

The Commonwealth Privacy Act and the Queensland Health Services Act 1991 impacts on release of some forms of data. **In particular, an individuals' identifiable data will not be released.**

CKD.QLD Conditions of Release of data

CKD.QLD provides data extracts to **external** groups for analysis under a series of conditions. Each request is considered on its merits by the CKD.QLD Management Committee, using the criteria set out in these guidelines for data release, and under the umbrella of Registry ethical conduct & approval.

Datasets provided for analysis are de-identified (ie names and personal identifiers are removed). In general, a specific data extract is created for each request, limited to the data needed to perform the analyses on the population requested. In general, groups requesting analysis data sets need to demonstrate capacity to perform appropriate statistical analysis, and also to have access to appropriate nephrological advice to interpret results. In most cases this will be achieved by the inclusion of nephrologists from contributing units as part of the collaboration.

Conditions of Use of data

Data is released to specific people for specific projects; use for other projects beyond the scope of the original application or release to other people or groups is not permitted without prior CKD.QLD Management approval. Security of the data is the responsibility of the researcher. It should be maintained in a secure computer storage facility.

Acknowledgement

If data from the CKD.QLD Registry is the primary source for a report or publication, a copy of this would ideally be sent to CKD.QLD prior to publishing. It is a condition of use of the CKD.QLD Registry that the source of the data is acknowledged, along with a statement that the analysis and interpretation are those of the author, not CKD.QLD (see Attribution of Publications below).

Attribution of Publications

Where a member of a participating unit has analysed data provided by CKD.QLD and subsequently prepared a manuscript, then "CKD.QLD Registry" should be acknowledged as a secondary institution in addition to the author's Centre, Hospital or University. This applies whether the primary data analysis is performed by the author or by CKD.QLD staff. Where the author is a CKD.QLD office holder or staff member then the primary attribution should be "CKD.QLD Registry".

Where CKD.QLD Registry data is only a minor portion of the work, then it may be more appropriate to acknowledge the source explicitly in the "Acknowledgements" section.

In both cases the disclaimer below should be included:

"The data reported here have been supplied by the CKD.QLD Registry. The interpretation and reporting of these data are the responsibility of the Authors and in no way should be seen as an official policy or interpretation of the CKD.QLD Registry."

In all cases the source and treatment of the data should be made clear in the "Methods" section. Preferably the abstract (and keywords if applicable) should also include "CKD.QLD" which would allow for searching Registry publications.

For any queries, please contact: Anne Cameron

Anne Cameron (Salisbury) RN I Manager: **NHMRC CKD.CRE** and **CKD.QLD** | Centre for Chronic Disease | The University of Queensland School of Medicine | Health Sciences Building level 8, Royal Brisbane & Women's Hospital, Herston QLD 4029 | Monday-Friday: ph +61 73346 4995 | fax +61 7 3346 5178 | email: anne.salisbury@health.qld.gov.au or a.salisbury1@uq.edu.au | www.ckdqld.org

Version 3.0 August 2016. Review date: August 2017.
ACKNOWLEGEMENT:
The ANZDATA Registry Data Access Guidelines where benchmarked in the development of this document.
